# Using Functional or Structural Magnetic Resonance Images and Personal Characteristic Data to Identify ADHD and Autism

**DOI:** 10.1371/journal.pone.0166934

**Published:** 2016-12-28

**Authors:** Sina Ghiassian, Russell Greiner, Ping Jin, Matthew R. G. Brown

**Affiliations:** 1 Department of Computing Science, University of Alberta, Edmonton, Alberta, Canada; 2 Department of Psychiatry, University of Alberta, Edmonton, Alberta, Canada; 3 Alberta Machine Learning Institute (AMII), formerly Alberta Innovates Centre for Machine Learning (AICML), Edmonton, Alberta, Canada; Aristotle University Of Thessaloniki Faculty of Forestry and Natural Environment, GREECE

## Abstract

A clinical tool that can diagnose psychiatric illness using functional or structural magnetic resonance (MR) brain images has the potential to greatly assist physicians and improve treatment efficacy. Working toward the goal of automated diagnosis, we propose an approach for automated classification of ADHD and autism based on histogram of oriented gradients (HOG) features extracted from MR brain images, as well as personal characteristic data features. We describe a learning algorithm that can produce effective classifiers for ADHD and autism when run on two large public datasets. The algorithm is able to distinguish ADHD from control with hold-out accuracy of 69.6% (over baseline 55.0%) using personal characteristics and structural brain scan features when trained on the ADHD-200 dataset (769 participants in training set, 171 in test set). It is able to distinguish autism from control with hold-out accuracy of 65.0% (over baseline 51.6%) using functional images with personal characteristic data when trained on the Autism Brain Imaging Data Exchange (ABIDE) dataset (889 participants in training set, 222 in test set). These results outperform all previously presented methods on both datasets. To our knowledge, this is the first demonstration of a single automated learning process that can produce classifiers for distinguishing patients vs. controls from brain imaging data with above-chance accuracy on large datasets for two different psychiatric illnesses (ADHD and autism). Working toward clinical applications requires robustness against real-world conditions, including the substantial variability that often exists among data collected at different institutions. It is therefore important that our algorithm was successful with the large ADHD-200 and ABIDE datasets, which include data from hundreds of participants collected at multiple institutions. While the resulting classifiers are not yet clinically relevant, this work shows that there is a signal in the (f)MRI data that a learning algorithm is able to find. We anticipate this will lead to yet more accurate classifiers, over these and other psychiatric disorders, working toward the goal of a clinical tool for high accuracy differential diagnosis.

## Introduction

Mental disorders impose huge personal costs to individual patients and their families as well as economic costs to society [1, 2]. Improved diagnostics for mental illnesses may lead to improvements in detection and treatment for mental disorders, thereby alleviating some of this burden. Here, we work toward improving automated diagnosis for mental illness using machine learning with structural magnetic resonance imaging (MRI) [3] and functional MRI [4] of the brain. The basic approach is to use such MRI data as input to a machine learning algorithm to create classifiers that can classify (diagnose) novel individuals as patients or healthy controls.

This was the goal of the 2011 ADHD-200 Global Competition [5–7]. For that competition, the ADHD-200 Consortium made available a large dataset of functional and structural MRI data from patients with ADHD and healthy controls—almost one thousand participants in total. Twenty-one teams competed in the competition to train classifiers to predict whether another participant was healthy or had ADHD. Input data for the diagnostic process included resting state fMRI (RS-fMRI), structural MRI and personal characteristic data. The competition teams demonstrated some success in diagnosing ADHD using this data. The team from our institution (University of Alberta) submitted the best-performing classifier, which achieved an accuracy of 62.5% on the competition’s holdout dataset [6, 8]. Work subsequent to the competition has improved the accuracy to 66.7% [9].

Researchers have also trained classifiers to classify autism versus healthy control status, using the Autism Brain Imaging Data Exchange (ABIDE) dataset [10–12]. The best result published to date with the ABIDE dataset is an accuracy of 60.0% over a baseline of about 53.6% [12].

The ADHD-200 and ABIDE datasets are large, each containing about 1000 participants (including both patients as well as healthy controls) from multiple institutions. Each includes both functional and structural MRI data from each participant, as well as other characteristics. One crucial advantage of using large, multi-site datasets is that they include a greater diversity of participants within and between sites. This is in contrast to smaller datasets of more homogeneous, often hand-picked, participants recruited from one site. The greater diversity of participants from a larger dataset makes it harder to obtain high accuracy results, but diagnostic systems that do well with a large dataset tend to be more reliable, robust and generalize better to new participants in comparison to systems honed to work on a small dataset from a single site [13–15].

The accuracy results cited above are less than 70%. They are not yet at the level of clinical utility but do provide justification for continued work on using learning techniques to produce automated diagnostic systems based on (f)MRI data. A number of other studies in this area have reported higher accuracies in diagnosing various psychiatric conditions (for examples, see [16–19]). However, these studies used much smaller datasets, typically containing fewer than 100 participants and often under 20 participants. The participants typically came from only a single site in these studies. Similarly, some groups have reported higher accuracies using small subsets of the ADHD-200 or ABIDE datasets (eg: [20–22]). One problem with using such small datasets, as opposed to the full ADHD-200 or ABIDE datasets, is that results are much less likely to generalize to new individuals due to the higher chance of over-fitting to a small dataset. This observation is supported by Katuwal et al. [15], who showed that it is possible to achieve much higher accuracy classifying patients vs. controls using smaller subsets of the ABIDE dataset in comparison to a larger subset of the ABIDE data. Generalization is obviously of critical importance for clinical utility.

As stated above, the large ADHD-200 and ABIDE datasets offer the advantage that their data were collected from multiple sites. Though this increases the variance in the data, and therefore the difficulty in learning high accuracy classifiers with such data, between-site variance is an important real-world phenomenon that any clinical learning/diagnostic tool must accommodate, as a classifier trained on a single dataset is likely to be less robust to diverse types of patients than one trained on many different datasets. In addition, if the learning process involves only a single dataset, the resulting (cross-validation) score will be overly optimistic—*i.e.*, it will not report the problems that will be encountered on these different types of patients. This is why we used almost all participants from the large ADHD-200 and ABIDE datasets, discarding as few participants as possible—removing only those participants whose data did not meet our quality assurance criteria. (See section Datasets.)

Image texture gives information about the spatial arrangement of intensities in an image or selected regions of an image [23]. Chang *et al.* [24] reported that morphological brain changes described by 3D texture analysis can be used to distinguish ADHD patients versus healthy controls. One way to describe the image texture is as the gradient vector’s angle and magnitude for each pixel/voxel of the image. Among the various algorithms that represent the image texture by gradient vectors, the histogram of oriented gradients (HOG) method has been used successfully in many different tasks [25]. We investigated the use of HOG texture features in functional and structural MR images for distinguishing healthy controls versus patients. Our hypotheses were that such MRI data contain information that can be used to differentiate individuals as either patients or healthy controls (*i.e.*, a two-class classification problem). This study describes our (f)MRI HOG-feature-based patient classification (MHPC) learning algorithm. MHPC learns an automated system for classifying ADHD from the ADHD-200 dataset, or for classifying autism from the ABIDE dataset, by using HOG features extracted from functional or structural MRI data as input to a collection of base machine learners. Our work makes two specific contributions: *a*) We show that HOG feature descriptors of either resting state fMRI or structural MRI data can be useful for classifying psychiatric disorders with accuracy above chance. *b*) The presented method outperforms all previously-published classification results for ADHD and autism using the two large resting-state fMRI/MRI datasets mentioned above.

## Methods

**Fig 1:**
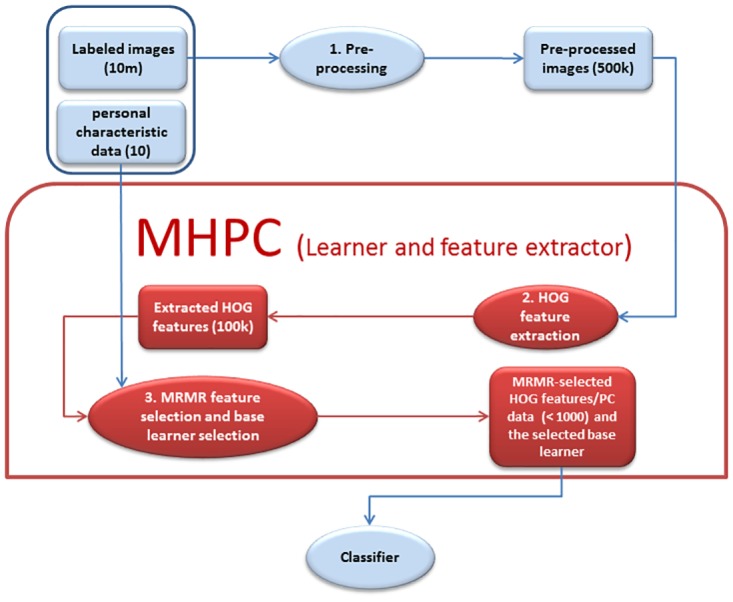
Summary of the learning pipeline. 1) Each image in the datasets is preprocessed (see section Preprocessing and Fig 2), reducing the dimensions from about 100,000,000 (79 × 95 × 68 × 200) to about 500,000. 2) The MHPC system then extracts the 3D-HOG features of each image reducing the number of dimensions to about 100,000; see section Histogram of oriented gradients (HOG) features. 3) The last step tries to select the best learner (from the initial set of base learners) and feature set, based on running 5-fold cross validation over the training set, using different combinations of the number of features and base learners. This step reduces the number of dimensions to a number under 1000; see section Results. HOG feature extraction, minimum redundancy maximum relevance (MRMR) feature selection and base learner selection are all parts of the MHPC algorithm (shown in the red box above). See Algorithm 1 for details. This figure is best viewed in color.

This section explains our MHPC learning algorithm in detail. The pipeline in Fig 1 summarizes how we processed the data to produce a classifier. Section Datasets gives information on the datasets on which we ran our MHPC learning algorithm. The section Preprocessing explains the preprocessing pipeline we used for functional and structural brain images. Section Histogram of oriented gradients (HOG) features explains briefly how HOG features work and how we extended them to work in the 3D space. Section Classifier for automated diagnosis describes how we used HOG features along with some machine learning tools to build a learning algorithm in order to produce classifiers capable of diagnosing healthy versus autism or healthy versus ADHD.

### Datasets

We analyzed datasets collected at other institutions and made publicly available on the internet for research purposes. All data were collected under the direction and approval of the respective institutions’ ethics boards. All participant data were anonymized by the collecting institutions prior to being made publicly available. We conducted a re-analysis of these existing, public, anonymized data in accordance with the policies of the University of Alberta’s Research Ethics Board and the Canadian Tri-Council as outlined in [26].

We used the ADHD-200 dataset [5–7]. This dataset includes resting state fMRI and T1-weighted structural MRI data from a total of 973 subjects, including patients with ADHD as well as healthy controls. Participant data was collected at eight different imaging sites, in North America, Europe and China. Individual diagnoses were based on the specific clinical criteria used at each of the eight institutions. For details, see [5]. The ADHD-200 Global Competition divided the ADHD-200 dataset into training and testing sets. We use those same sets here. The ADHD-200 *training* dataset includes 776 subjects from seven different imaging sites. 491 subjects are healthy controls, while the other 285 subjects are patients with ADHD. The ADHD-200 *test* dataset is comprised of 197 subjects, including both healthy controls and patients with ADHD from seven different sites. (Note that the entire ADHD-200 dataset, including training and test data, was collected from eight different imaging sites. See [5–7] for details. One site, Brown University, was not present in the training data, and one different site, Washington University, was not present in the test data.) We had to remove seven of the training set subjects since six had no resting-state scan and one could not be preprocessed using our preprocessing pipeline (see below). We removed 26 test set subjects from the Brown University site as they had no diagnostic labels. We were left with 490 healthy controls and 279 ADHD patients in the training set and 94 healthy controls and 77 ADHD patients in the test set.

The ADHD-200 dataset also includes non-imaging, personal characteristic features for each subject. Full details are provided in [5, 6, 8]. We excluded features that were directly related to the diagnosis, such as questionnaires that measured severity of ADHD symptoms and medication status. We also excluded measures for which 30% or more of participants had missing values, specifically, Verbal IQ, Performance IQ, and Full2 IQ. We used the following personal characteristic features: sex, age, handedness, site of imaging, IQ Measure and Full4 IQ. Values for IQ Measure and Full4 IQ were missing in some subjects (less than 30% of subjects). In these cases, we replaced each missing value with the mean of the values we had available for this feature. For additional details, see Tables 1 and 2 as well as [5, 6, 8].

**Table 1:** Details of Training Dataset participants, ADHD-200.

Group	N	Age(years)	Full 4 IQ	Sex (%)		Handedness (%)		
				F	M	Right	Left	Ambi.
Healthy Controls	490	12.2 ± 3.3	114 ± 13	47	53	97	3	0.2
Patients w/ ADHD	279	11.6 ± 2.9	107 ± 13	21	79	97	3	0.4

**Table 2:** Details of Holdout Dataset participants, ADHD-200.

Group	N	Age(years)	Full 4 IQ	Sex (%)		Handedness (%)		
				F	M	Right	Left	Ambi.
Healthy Controls	94	11.9 ± 4.2	114 ± 11	49	51	95	5	0
Patients w/ ADHD	77	11.6 ± 3.2	107 ± 12	22	78	96	4	0

We also explored the Autism Brain Imaging Data Exchange (ABIDE) dataset [10]. This dataset is comprised of resting state fMRI and T1-weighted structural MRI scans from 1112 subjects, including patients with autism and healthy controls. ABIDE participant data was collected at 17 different institutions in North America and Europe. Details of the diagnostic criteria used at the different institutions are provided in [10]. We had to exclude one subject whose data could not be preprocessed with our preprocessing pipeline, leaving us with a dataset of 1111 individuals, including 573 healthy controls and 538 patients with autism. Unlike the ADHD-200 dataset, the ABIDE dataset does not have official training and test sets specified by the data curators. Therefore, we divided the ABIDE dataset into training and test sets by randomly selecting a label-balanced 889 (4/5) of the ABIDE subjects as the training set, leaving the remaining 222 (1/5) ABIDE subjects as the testing set.

The ABIDE dataset also provided an extensive array of non-imaging, personal characteristic information from which we used age, sex, handedness, full scale IQ, verbal IQ, performance IQ, site of the imaging and eyestat (which indicated whether the person kept his eyes open or not during the scan). For the ADHD-200 dataset, we excluded features that were directly related to the diagnostic label, as well as features that were missing for 30% or more of the participants. For personal characteristic features with missing values, we filled in missing values with the mean of the non-missing values for the given feature. For more information on this dataset, see Tables 3, 4 and [10].

**Table 3:** Details of Training Dataset participants, ABIDE.

Group	N	Age(years)	P IQ	F IQ	V IQ	Sex (%)		Handedness (%)		
						F	M	Right	Left	Ambi.
Healthy Controls	458	17.1 ± 7.9	108 ± 12	111 ± 12	111 ± 12	17	83	93	5	2
Patients w/ Autism	430	17.3 ± 8.4	105 ± 15	106 ± 16	105 ± 16	11	89	89	8	3

**Table 4:** Details of Holdout Dataset participants, ABIDE.

Group	N	Age(years)	P IQ	F IQ	V IQ	Sex (%)		Handedness (%)		
						F	M	Right	Left	Ambi.
Healthy Controls	115	16.7 ± 6.7	107 ± 13	109 ± 12	110 ± 13	18	82	93	6	1
Patients w/ Autism	108	16.6 ± 7.9	103 ± 18	103 ± 17	102 ± 17	17	83	94	5	1

Handedness scores from all sites except the NeuroIMAGE site (ADHD-200 dataset) were categorical. We modified the NeuroIMAGE handedness scores to fit the categorical scheme by replacing all positive scores by 1 (right-handed) and all negative values with 0 (left-handed). We then used categorical handedness data as an input feature for the diagnosis task in some of the analyses as detailed below.

Each of the datasets included a high resolution T1-weighted structural MRI scan, as well as one or more resting-state functional MRI scans for each of the subjects. The functional MRI scans included 76 to 261 time points for each subject in the ADHD-200 dataset and 82 to 320 time points for the ABIDE dataset. Different subjects were scanned with different temporal resolutions (*i.e.*, sampling period or volume time), ranging from 1.5 s through 3 s in the ADHD-200 dataset and from 1 s through 3 s in the ABIDE dataset. For further details of the MRI scanning protocols used in both datasets see [5, 10].

There are many challenges in processing such multisite datasets that are not present in datasets gathered in a single site, including site-specific range of intensity values, different scanning durations, different volume times and other batch effects. Our preprocessing pipeline (see section Preprocessing) therefore included steps to normalize various aspects of the data across different sites.

### Preprocessing

For preprocessing, we used SPM8, a software package designed for analyzing brain imaging data and also for preprocessing fMRI data [27, 28] and our own in-house MATLAB code.

**Fig 2:**
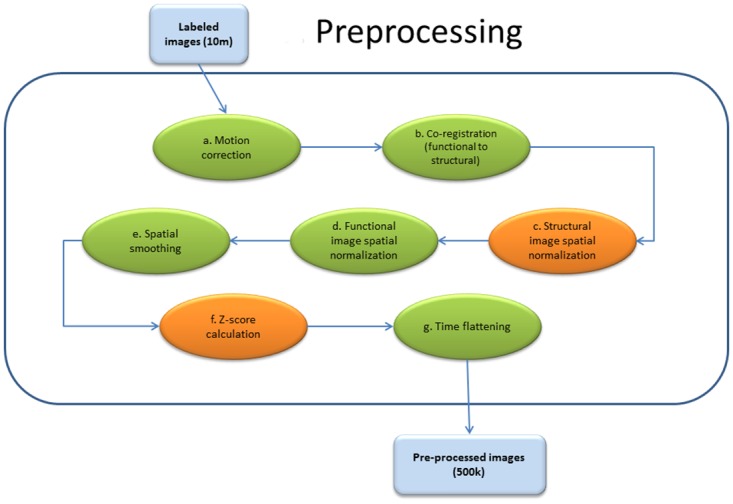
Preprocessing pipeline. The preprocessing pipeline for functional and structural magnetic resonance images is summarized in the figure. Orange shapes in the image show the steps of preprocessing necessary for both fMRI and structural MRI scans. Green shapes show the preprocessing steps only needed for fMRI scans. This figure is best viewed in color.

In our preprocessing pipeline, we used standard methods from the structural MRI and fMRI literature. As shown in Fig 2, our fMRI preprocessing involved seven steps, two of which are also used for MRI: *a*) 6-parameter rigid body motion correction of functional scans, *b*) co-registration of functional scans to subject-specific structural scans to guide the spatial normalization step, *c*) non-linear spatial normalization (parameter estimation and spatial transformation) of structural images to the MNI T1 template [29–31], *d*) non-linear spatial normalization of previously co-registered functional image volumes to MNI T1 template using warping parameters computed in the structural image normalization, *e*) spatial smoothing of functional image volumes with 8 mm full width half maximum (FWHM) Gaussian kernel [4], *f*) to standardize the intensities of images scanned from different sites, we replaced each entry with its subject-based z-score. For functional images, this means computing the z-score over the whole 4 dimensional image, for each subject separately. For structural images, we computed the z-score over the whole 3 dimensional image for each subject. Finally we performed a seventh step for functional data: *g*) averaging of functional scans across time to make a single time point functional image for each subject.

In the spatial normalization step (step *c*), we used a bounding box of [−78, −112, −50] to [78, 76, 85], which are the SPM8 defaults, and a voxel size of 2 by 2 by 2 millimeters. In the z-normalization step (step *f*), we computed the mean and the standard deviation over all voxel values in a given structural image or all voxel values in all volumes in a given functional scan (*i.e.*, all voxel values for a specific subject, over all time points for fMRI data) and then subtracted the mean of the image from each voxel’s value and then divided the resulting value by the standard deviation.

An fMRI scan is four dimensional with three spatial dimensions and one time dimension: *f*(*x*, *y*, *z*, *t*) is the intensity value of the voxel at location (*x*, *y*, *z*) and at time *t*. We reduced the number of dimensions in the fMRI scans to three (step *g*) by setting the value for each (*x*, *y*, *z*) location to the average value:

$$\begin{array}{c} f\left(x,y,z\right)\;=\;\frac{1}{k}\sum_{t=1}^{k}f\left(x,y,z,t\right) \end{array}$$

where *k* is the number of time points during the scan. This produced what we call a 3D functional MR image for each individual.

A summary of our preprocessing pipeline for fMRI and also structural MRI scans can be found in Fig 2.

### Histogram of oriented gradients (HOG) features

In this section we describe how histogram of oriented gradients work and explain why we expect them to be useful for functional and structural MRI scans. One should refer to the original paper [25] to thoroughly understand how and why these features are good for different purposes. Below, we define HOG features for three-dimensional space—this requires some changes from the original HOG description, which was designed to work for two-dimensional space.

#### 3D HOG

**Fig 3:**
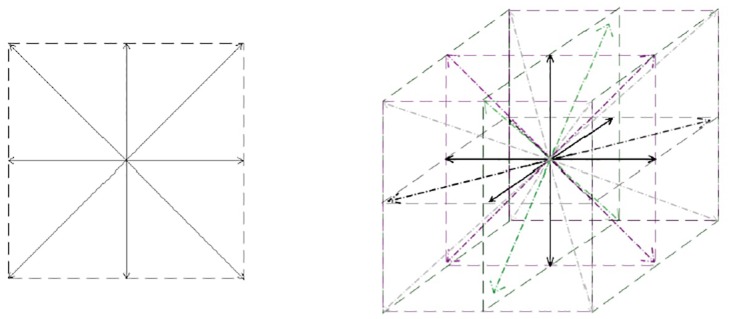
HOG bins in 2D and 3D space. Left and right panels show HOG bins in 2D and 3D space, respectively.

The idea behind the histogram of oriented gradient (HOG) descriptors is that the intensity gradients’ distribution can describe the object appearance and shape [25]. The input of the HOG algorithm is an image along with the size of each cell (how many pixels/voxels should a cell contain), the size of the blocks (how many cells should a block contain), and the number of bins for each cell (see Fig 3). The output is a histogram over the specified number of bins for each cell of the image. The left and right panels show 2D and 3D HOG bins, respectively.

The HOG feature extraction algorithm divides the whole image into blocks and each block into several cells, where each cell involves a set of pixels (note that a cell can belong to multiple blocks). In each cell, it utilizes the local histograms of gradient orientations as a new feature. It then normalizes each cell within different blocks. This can be done by considering overlapping blocks through the image (see [25] for a thorough explanation). Each cell then contributes to the final feature vector a few times, normalized within different blocks.

Given the function *f*(*x*, *y*, *z*), that maps each (*x*, *y*, *z*) position in a 3D image to its intensity value, we have the derivative:

$$\begin{array}{c} \nabla f\left(x,y,z\right)\;=\;[\begin{array}{c} \frac{\partial f(x,y,z)}{\partial x}\\ \frac{\partial f(x,y,z)}{\partial y}\\ \frac{\partial f(x,y,z)}{\partial z} \end{array}]\;=\;[\begin{array}{c} f_{x}\\ f_{y}\\ f_{z} \end{array}] \end{array}$$

where:

$$\begin{array}{c} f_{x}\left(x,y,z\right)\;=\;\frac{\partial f(x,y,z)}{\partial x}\;\approx \;\frac{f(x+1,y,z)-f(x-1,y,z)}{2} \end{array}$$

$$\begin{array}{c} f_{y}\left(x,y,z\right)\;=\;\frac{\partial f(x,y,z)}{\partial y}\;\approx \;\frac{f(x,y+1,z)-f(x,y-1,z)}{2} \end{array}$$

$$\begin{array}{c} f_{y}\left(x,y,z\right)\;=\;\frac{\partial f(x,y,z)}{\partial z}\;\approx \;\frac{f(x,y,z+1)-f(x,y,z-1)}{2} \end{array}$$

The gradient magnitude is:

$$\begin{array}{c} \left|\nabla f\left(x,y,z\right)\right|=\sqrt{f_{x}^{2}+f_{y}^{2}+f_{z}^{2}} \end{array}$$

The HOG algorithm also finds the bin with the maximum overlap with the gradient vector as follows:

$$\begin{array}{c} \alpha \left(\nabla f,b\right)\;=\;\frac{\nabla f(x,y,z)\cdot b}{\left|\nabla f(x,y,z)|\times |b\right|} \end{array}$$

$$\begin{array}{c} \text{dir}\left(x,y,z\right)\;=\;\underset{b}{\text{argmax}}\;\alpha \left(\nabla f,b\right) \end{array}$$

where *b* ranges over each of the 26 vectors (see Fig 3). Then this *b* vector contributes to its corresponding bin. For each voxel at location (*x*, *y*, *z*), HOG first computes a direction dir(*x*, *y*, *z*), and then increments the associated bin:

$$\begin{array}{c} {\text{bin}}_{\text{dir}(x,y,z)}+=\left|\nabla f\left(x,y,z\right)\right| \end{array}$$

**Fig 4:**
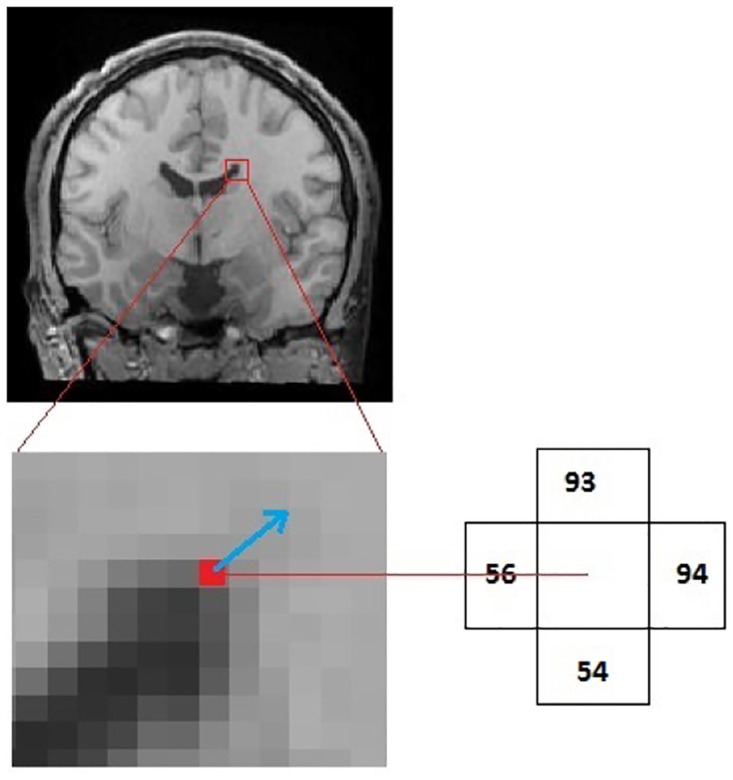
Gradient vector of a sample pixel. For illustration, we describe the 2D HOG feature computation process. Here, we consider a single pixel, the one shown in red, whose neighbors have intensities 56, 93, 94, and 55. The blue arrow is the sample gradient, computed as described below. (This figure is best viewed in color.)

**Fig 5:**
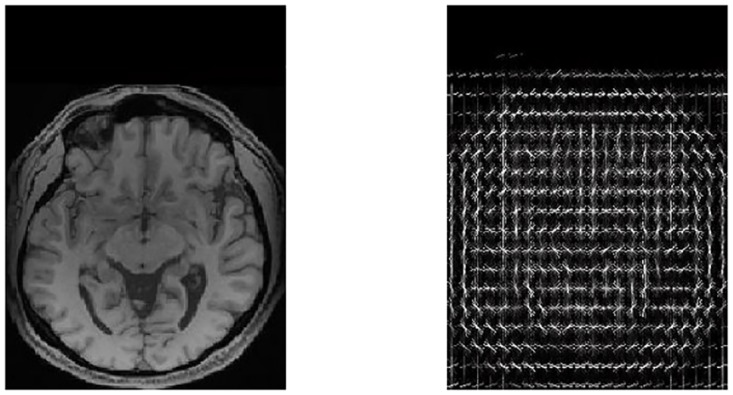
Input and output of 2D HOG on a brain image. The left panel shows an axial slice of a structural MR image of a brain. The right panel shows the HOG features of the same slice. Here, we represent the HOG features by an 8-sided “star”, where the length of each arm is the size of the histogram in that direction. This representation is generated using VLFeat [32].

To better understand how HOG features are computed, a 2*D* version of this is illustrated in Fig 4. See Fig 5 for the representation of HOG features used on a 2D brain image slice.

$$\begin{array}{c} \nabla f\left(x,y\right)=[\begin{array}{c} \frac{\partial f(x,y)}{\partial x}\\ \frac{\partial f(x,y)}{\partial y} \end{array}]=[\begin{array}{c} \frac{94-56}{2}\\ \frac{93-54}{2} \end{array}]=[\begin{array}{c} 19\\ 19.5 \end{array}] \end{array}$$

$$\begin{array}{c} \text{Magnitude}=\sqrt{{\left(19\right)}^{2}+{\left(19.5\right)}^{2}}=27.22 \end{array}$$

Then to find the maximum overlapping bin we compute:

$$\begin{array}{c} \text{dir}\left(x,y\right)=\underset{b}{\text{argmax}}\;\alpha \left(\nabla f\left(x,y\right),b\right) \end{array}$$

where b ranges over the angles shown in Fig 3. The biggest *α* is produced when b* = (1, 1):

$$\begin{array}{c} \alpha \left(\nabla f\left(x,y\right),b^{*}\right)=\frac{\nabla f\left(x,y\right)\cdot b^{*}}{\left|\nabla f\left(x,y\right)|\times \right|b^{*}|}=\frac{\left(19,19.5)\cdot (1,1\right)}{27.22\times \sqrt{2}}\approx 1 \end{array}$$

$$\begin{array}{c} \text{dir}(x,y)=(1,1) \end{array}$$

$$\begin{array}{c} {\text{bin}}_{\left(1,1\right)}+=27.22 \end{array}$$

For each cell of many voxels, HOG will build a histogram; it will then concatenate the cell histograms in each block into a single vector, which is then normalized. Here, as each block includes 2 × 2 × 2 = 8 cells, we identify each block with 8 × 26 = 208 values. Here, let *v* represent the histogram, viewed as the tuple of values in a block. One of the successful normalization schemes used is [25]:

$$\begin{array}{c} v\to \frac{v}{\sqrt{{\left||v|\right|}_{2}^{2}+\epsilon^{2}}} \end{array}$$

where ||*v*||_2_ is the 2-norm and *ϵ* is a small constant, which helps in cases where all the gradient vector’s magnitudes are equal to zero in a block. A thorough explanation of different block normalization schemes can be found in the original paper [25]. 3D HOG, with these parameters, identifies each subject with 116,480 features.

HOG has been successfully applied to 2D images for various tasks related to object recognition [25]. Here we explore whether this successful method can detect any differences in healthy control brains and non-healthy brains to diagnose different diseases.

### Classifier for automated diagnosis

Given a training dataset *D* that includes *N* training examples each with *d* features where each training example belongs to one of two possible classes (healthy or patient), the goal of learning is to find a function *C*: *X* → *Y* where *X* is the feature space and *Y* is the output domain—*i.e.*, *C* maps each example in *X* to one of the possible classes. Here,

$$\begin{array}{c} X\subseteq R^{d},\;Y=\left\{0,1\right\} \end{array}$$

$$\begin{array}{c} D\subseteq {\left(X\times Y\right)}^{N} \end{array}$$

$$\begin{array}{c} L(D)\to C \end{array}$$

In general, a learner *L* learns a classifier *C* based on the data available in dataset *D*. Here, we consider the MHPC learner, which uses the HOG features of labeled brain images and personal characteristic data (over a large number of instances) as input, to learn two-class classifiers to diagnose either ADHD versus control (using the ADHD-200 data) or autism versus control (using the ABIDE data). MHPC considers several base learners (support vector machines with linear kernel, support vector machines with radial basis function kernel and a specific sigma value, decision trees, k-nearest neighbours and naive bayes [33]), the 3*D* HOG feature extraction method [25] and the minimum redundancy maximum relevance (MRMR) feature selection algorithm [34, 35]. MHPC returns the best classifier over a subset of HOG features of the images and perhaps other patient features, based on a 5-fold cross validation on the training set. As each dataset had around 1000 individuals only, there was a high chance that the learning algorithms would overfit to the training data if we used all of the features. We therefore used MRMR (maximum relevance minimum redundancy) [34, 35] as a preprocessing step to select the most relevant features (Fig 1 part 3). The MRMR feature selection method sequentially selects the features that are most relevant to the class variable (high mutual information) and minimizes the redundancy of the selected features. For a thorough explanation of MRMR feature selection algorithm, see [34, 35].

Our MHPC system is summarized in Algorithm 1. For notation, let:

- acc(L, D, FS) be the 5 values computed from the folds of 5-fold cross validation, using base learner L on dataset D with feature set FS,
- Eacc(L, D, FS) be the mean of these 5 values,
- Racc(L, D, FS) to be the range of accuracy values over the 5 folds (see the range variable of line 7 in Algorithm 1),
- D_train_ be the training data, and D_test_ the hold-out data,
- FS_k_(D) be the top k MRMR features over the dataset D,
- LS be the set of base learners: LS = { SVM-linear, SVM-RBF-1, SVM-RBF-2, …, SVM-RBF-9, NB, Decision Tree, KNN },
- SVM-RBF-i denote support vector machine with radial basis function kernel and sigma value equal to i,
- L* denote the best base learner and
- FS*(L*) denote the best feature set associated with the best learner.

**Algorithm 1:** MHPC (see section Classifier for automated diagnosis for acronyms)

1: **procedure** MHPC(D: training data, L: set of base learners)

2:  **for each** base learner L in L **do**

3:   FS ← top 1 MRMR feature (on D)

4:   **repeat**

5:    vals[L, 1: 5] ← acc(L, D, FS)

   //*5-fold cross validation accuracy of L on D, using only the features FS*

6:    mean ← ave_i_ {vals[L, i]}

7:    range ← max_i_{vals[L, i]} − min_j_{vals[L, j]}

   //*range of accuracy over the 5 folds*

8:    AveAcc[L, FS] ← [mean, range]

9:    FS ← FS + top 1 new MRMRSelectedFeatures from D

10:   **until** (No accuracy increase in all of the learners)

**Fig 6:**
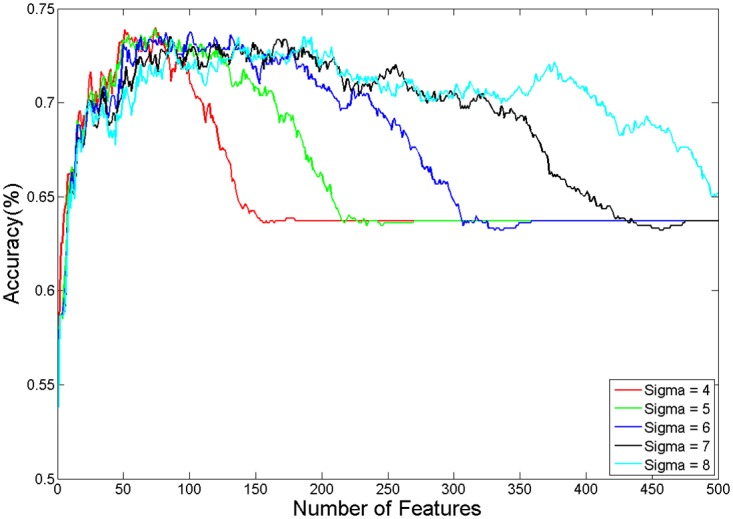
5-fold cross validation accuracies on the training set. The accuracies are obtained using RBF SVM (with various sigma values), on the training portion of the ADHD-200 dataset using functional images plus personal characteristic data. This figure is best viewed in color.

   // *see text and* Fig 6 *for more information*

11:  **end for**

12:  i ← 1

13:  **for** **each** base learner L in L **do**

14:   $\text{bestFS}\leftarrow \underset{\text{FS}}{\text{argmax}}\{\text{AveAcc}[\text{L, FS}][1]\}$

 // *the feature set that has the largest mean value for this learner L*

15:   TopAccs[i] ←

    [L, bestFS, AveAcc[L, bestFS][1], AveAcc[L, bestFS][2]]

 //*this tuple lists the best feature set for this L, along with the average accuracy for this [L, bestFS] pair, and the range of accuracies*

16:   i ← i + 1

17:  **end for**

18:  TopAccs ← sort TopAccs corresponding to third element

 // *which is the mean value of each [L, FS] pair, sorted large to small*

19:  BestAcc [1: 5] ← TopAccs [1: 5]

20:  BestL ← tuple in BestAcc with smallest 4th entry (range)

21:  L* ← BestL [1, 1]

22:  FS* ← BestL [1, 2]

23:  classifier ← L*(D, FS*)

 //run learner L* on whole training set D, using only the featuresFS*

24:  **return** classifier

25: **end procedure**

Our MHPC system considers various base learners (with various parameters). Using dataset D (here we used D_train_ only), for each base learner L ∈ LS, MHPC sequentially considers using the top k = {1, 2, 3, …} MRMR features. It uses a 5-fold cross validation approach, stopping when there is no accuracy increase in any of the base learners and the Eacc(L, D, FS_k_) reaches a plateau. Fig 6 shows that each of the learners reaches a peak and then drops significantly after that point. We identified each base learner with both the mean accuracy achieved using the best feature set, Eacc(L, D_train_, FS) and also Racc(L, D_train_, FS) on D_train_. We found the 5 base learners with the top 5 mean accuracy values. Because the top 5 accuracies were very close, our MHPC chose the learner with the smallest Racc(L, D_train_) and returned that base learner, L*. Note that all determinations of classifier algorithm choice, feature selection, parameter values, and trained classifier weight values were made using only the training set data D_train_. After learning this L* and FS*(L*), we then ran the base learner L* with feature set FS*(L*) on the test set D_test_. Testing on the hold-out test set D_test_ protected against overfitting in terms of algorithm choice, feature selection, parameter values, and learned weight values. The performance results on the test sets for the ADHD and ABIDE datasets constitute the primary measures of our MHPC system’s performance. For illustrative purposes, we also report certain results from the learning process (that is, results from the training sets). Due to issues with overfitting, results from the training sets are included only to highlight aspects of the learning process.

## Results

The ADHD-200 Global Competition divided the ADHD-200 dataset into training and test sets; we used this split. In the ABIDE dataset, we randomly selected a label-balanced 4/5 of the data as the training data, and left the remaining 1/5 as the testing data. We ran our MHPC system on each training dataset using different kinds of training and testing data that varied based on whether it included personal characteristic and/or RS-fMRI and/or structural MRI features. (Below we consider five of the 2^3^ = 8 sub-collections of these three types of features). We ran MHPC on various sub-collections of these feature sets—in each case, following the methodology mentioned above.

Below, section Functional images represents the results of the learning using only RS-fMRI. Section Structural images explains the results of using only structural MRI scans. The results of using only personal characteristic data can be found in section Personal characteristic data. When we combined personal characteristic data with functional or structural images, we obtained the results listed in section Adding personal characteristic data to functional or structural images.

### Functional images

We ran our MHPC algorithm using the 116,480 HOG features derived from only the fMRI data from the ADHD-200 (respectively ABIDE) dataset. For the ADHD-200 training dataset, MHPC determined that the best learner was the RBF SVM with Sigma = 9 and a specific set of 469 HOG features. When we ran this learning and feature set on the test set, its accuracy was 59.7%. For the ABIDE dataset, MHPC decided that SVM with RBF kernel with Sigma = 6 with 110 features was the best; when it was run on its hold-out set, its accuracy was 59.2%.

Tables 5 and 6 list the top five learners, their accuracy on the training set using 5-fold cross validation and the best learner’s accuracy on the test set. When using only functional images we found that all top five learners were support vector machines with RBF kernels, with different Sigma values. In all of the tables, the learners are sorted in decreasing value of the training accuracy.

**Table 5:** ADHD-200, functional images.

Learner^+^, L	Number of features, |FS*(L)|	Training Accuracy, Eacc(L, D_train_, FS*(L))	Range	Test Accuracy, acc(L*, D_test_, FS*(L*))
RBF-9	469	70.4%	7.4%	59.7%
RBF-8	311	70.2%	11.3%	
RBF-7	227	69.3%	11.0%	
RBF-6	204	69.3%	11.9%	
RBF-5	145	68.9%	9.1%	

^+^RBF-i represents SVM with RBF kernel with Sigma = i. (This is true for all subsequent tables as well). For definitions of mathematical symbols, see section Classifier for automated diagnosis.

**Table 6:** ABIDE, functional images.

Learner, L	Number of features, |FS*(L)|	Training Accuracy, Eacc(L, D_train_, FS*(L))	Range	Test Accuracy, acc(L*, D_test_, FS*(L*))
RBF-8	128	58.9%	5.3%	
RBF-9	248	58.9%	7.3%	
RBF-3	53	58.8%	8.4%	
RBF-5	110	58.6%	3.9%	
RBF-6	110	58.6%	2.5%	59.2%

### Structural images

We then explored the performance using structural MRI (structural images)—that is, just using T1-weighted images. We ran the same processing, including HOG feature extraction, MRMR feature selection, and using the same set of base learners. The results for both datasets are shown in Tables 7 and 8.

**Table 7:** ADHD-200, structural images.

Learner, L	Number of features, |FS*(L)|	Training Accuracy, Eacc(L, D_train_, FS*(L))	Range	Test Accuracy, acc(L*, D_test_, FS*(L*))
RBF-8	285	72.3%	5.2%	66.1%
RBF-9	467	72.2%	6.5%	
RBF-7	274	71.7%	7.6%	
RBF-6	204	71.5%	7.6%	
RBF-5	131	70.9%	7.0%	

**Table 8:** ABIDE, structural images.

Learner, L	Number of features, |FS*(L)|	Training Accuracy, Eacc(L, D_train_, FS*(L))	Range	Test Accuracy, acc(L*, D_test_, FS*(L*))
RBF-9	590	58.9%	7.6%	
Naive Bayes	11	58.7%	4.5%	
RBF-6	194	58.1%	4.2%	60.1%
RBF-7	357	58.1%	10.9%	
RBF-8	651	57.9%	8.1%	

Here we found that the top five learners for the ADHD-200 dataset were all SVM with RBF kernels while in the ABIDE dataset MHPC chose Naive Bayes as one of the top five learners, although the Naive Bayes learner was not selected as the best. The test accuracy in the ADHD-200 dataset was 66.1% using RBF SVM-8 with 285 features. The ABIDE test set diagnosis accuracy was 60.1% using RBF SVM-6 with 194 features.

### Personal characteristic data

We also investigated whether personal characteristic data (i.e. non-imaging data) can help the classification of these psychiatric disorders. For the ADHD-200 dataset, we used age, sex, handedness, IQ measure, full4IQ score and site of the imaging. For the ABIDE dataset, we used age, sex, handedness, fIQ standard score, pIQ standard score, vIQ standard score, site of the imaging and eyestat. (See section Datasets for details.)

Using the MHPC algorithm with only personal characteristic data as the input for classifying the ADHD disease gave an accuracy of 69.0% (over baseline = 55.0%), which is consistent with the result of [8]. The same process with the ABIDE dataset resulted in 59.6% accuracy over the baseline of 51.6%.

In the ADHD-200 dataset, our MHPC system selected SVM with RBF kernel with Sigma = 1 and five features as the best learner. The only feature that was not chosen by our algorithm was IQ measure. In the ABIDE dataset, our method chose SVM with RBF kernel with Sigma = 2 as the best learner, and all eight features. The MHPC-chosen learners and the test accuracies of both datasets are shown in Tables 9 and 10.

**Table 9:** ADHD-200, personal characteristic data.

Learner, L	Number of features, |FS*(L)|	Training Accuracy, Eacc(L, D_train_, FS*(L))	Range	Test Accuracy, acc(L*, D_test_, FS*(L*))
RBF-1	5	71.8%	5.8%	69.0%
RBF-2	4	70.8%	9.7%	
Naive Bayes	6	70.7%	10.0%	
RBF-3	4	70.2%	10.4%	
RBF-4	5	70.0%	7.1%	

**Table 10:** ABIDE, personal characteristic data.

Learner, L	Number of features, |FS*(L)|	Training Accuracy, Eacc(L, D_train_, FS*(L))	Range	Test Accuracy, acc(L*, D_test_, FS*(L*))
Naive Bayes	8	62.7%	6.5%	
RBF-2	8	61.9%	3.4%	59.6%
RBF-6	7	61.9%	7.7%	
RBF-3	8	62.6%	6.0%	
RBF-4	8	62.1%	6.5%	

### Adding personal characteristic data to functional or structural images

We produced a training set by concatenating the personal characteristic features onto either the functional image features or the structural image features. For example, in the ADHD-200 dataset, we used 116,480 HOG features extracted from each functional image plus six personal characteristic data features to produce a 116,486-sized feature set. We then let MHPC choose which features to use for classification.

Brown *et al.* [8] showed that a learned classifier that uses only personal characteristic data without any imaging data can diagnose ADHD with an accuracy higher than any of the other approaches used in the ADHD-200 competition. Note that these other approaches used both functional images and personal characteristic data for classification and had structural images available for all of the subjects. The best two-class imaging-based classification accuracy achieved in the ADHD-200 competition was 61.5% when trying to build a two way classifier for healthy control status versus ADHD [6]. As described further above, we found that when using personal characteristic data alone, we could classify ADHD versus healthy control status with an accuracy of 69.0%, which is 14.0% above the baseline change accuracy of 55.0%. We also found that, with only personal characteristic data, we could classify autism with 59.6% accuracy, in contrast to 51.6% chance accuracy. We also investigated whether combining personal characteristic data with imaging data can improve classification of ADHD or autism.

**Fig 7:**
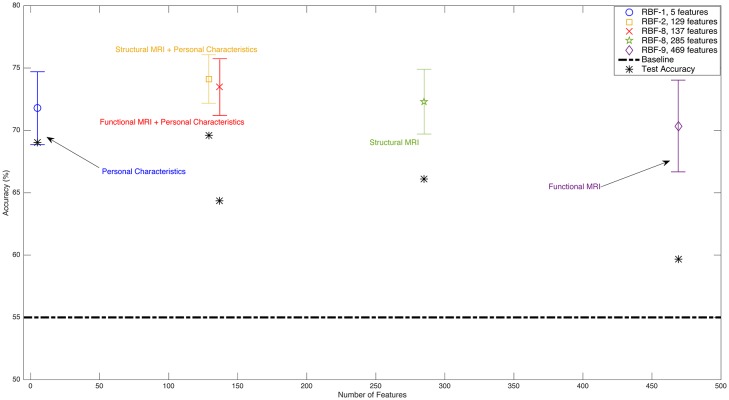
Summary of ADHD-200 dataset classification results. The black horizontal dotted line shows the baseline chance accuracy of the test set. Each vertical bar shows the mean and range of the cross validation results for the selected base learner (L) and feature set (FS*(L)) on the training set, as produced with MHPC (Algorithm 1). The blue asterisks * show the accuracy of each classifier on the hold-out set. The classifiers on the x-axis are ordered by the types of features they used, including various combinations of structural MRI, functional MRI, and personal characteristic data. The legend also identifies the actual classifier used. This figure is best viewed in color.

Fig 7 and Tables 5, 9 and 11 show that the accuracy of the learned classifier using only personal characteristic data is 69.0% and using only functional images is 59.7%, while the accuracy of using personal characteristic data with functional images is 64.3% on the ADHD-200 dataset. When we only use structural images as the input of our system, we achieve an accuracy of 66.1%. When we add personal characteristic data to structural images, the accuracy increases to 69.6% (see Tables 7 and 12). Comparing Tables 5 and 9 with 11, we observe that combining personal characteristic data and functional image data from the ADHD-200 dataset improves the classification performance compared to using only functional image data. Using only personal characteristic data results in better performance than using either functional image data alone or the combination of personal characteristic data and functional image data. This result is also consistent with Brown *et al.*’s results [8], as they too achieved better accuracy using personal characteristic data than functional image data. (Brown *et al.* [8] did not test the combination of both personal characteristic and functional image data.)

**Table 11:** ADHD-200, personal characteristic data with functional images.

Learner, L	Number of features, |FS*(L)|	Training Accuracy, Eacc(L, D_train_, FS*(L))	Range	Test Accuracy, acc(L*, D_test_, FS*(L*))
RBF-4	74	74.0%	8.4%	
RBF-5	73	73.9%	9.1%	
RBF-6	101	73.7%	6.3%	
RBF-7	85	73.5%	7.8%	
RBF-8	137	73.5%	4.6%	64.3%

**Table 12:** ADHD-200, personal characteristic data with structural images.

Learner, L	Number of features, |FS*(L)|	Training Accuracy, Eacc(L, D_train_, FS*(L))	Range	Test Accuracy, acc(L*, D_test_, FS*(L*))
RBF-8	83	74.5%	7.1%	
RBF-9	127	74.5%	6.5%	
RBF-3	42	74.5%	6.0%	
RBF-7	117	74.1%	7.8%	
RBF-5	129	74.1%	3.9%	69.6%

**Fig 8:**
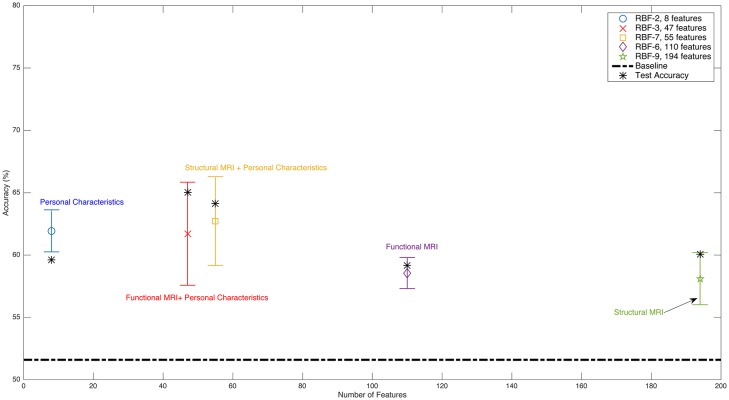
Summary of ABIDE dataset classification results. Conventions are the same as for Fig 7. This figure is best viewed in color.

On the other hand, in the ABIDE dataset (Tables 6, 10 and 13 and Fig 8), we see that the accuracy of using personal characteristic data as input is 59.6%, and the accuracy when using functional images is 59.2%. However, when we use these data together, we achieve over 65.0% accuracy. The same phenomenon happens for personal characteristic data and structural images (see Tables 8 and 14). Diagnostic classification accuracy for autism using structural MRI data is 60.1%, while the combination of personal characteristic data and structural MRI data yielded 64.1% accuracy. The results of all of the experiments are summarized in Fig 7 (ADHD-200 dataset) and Fig 8 (ABIDE dataset). Tables 15 and 16 summarize the accuracy, specificity and sensitivity for all of the learners (tested on the relevant hold-out test set).

**Table 13:** ABIDE, personal characteristic data with functional images.

Learner, L	Number of features, |FS*(L)|	Training Accuracy, Eacc(L, D_train_, FS*(L))	Range	Test Accuracy, acc(L*, D_test_, FS*(L*))
RBF-7	50	61.8%	8.6%	
RBF-3	47	61.7%	8.3%	65.0%
RBF-4	56	61.6%	9.0%	
RBF-6	39	61.5%	10.1%	
RBF-2	32	61.2%	9.3%	

**Table 14:** ABIDE, personal characteristic data with structural images.

Learner, L	Number of features, |FS*(L)|	Training Accuracy, Eacc(L, D_train_, FS*(L))	Range	Test Accuracy, acc(L*, D_test_, FS*(L*))
RBF-9	5	63.0%	8.4%	
RBF-4	54	62.9%	10.6%	
RBF-6	66	62.7%	9.9%	
RBF-7	55	62.7%	7.1%	64.1%
RBF-8	90	62.6%	8.2%	

**Table 15:** ADHD-200 results summary.

Data Source(s)	Accuracy	Sensitivity	Specificity
Functional MRI	59.7%	84.0%	29.9%
Structural MRI	66.1%	75.5%	54.5%
Personal Characteristic	69.0%	67.0%	71.4%
Structural + Personal Characteristic	69.6%	79.8%	57.1%
Functional + Personal Characteristic	64.3%	71.3%	55.8%

**Table 16:** ABIDE results summary.

Data Source(s)	Accuracy	Sensitivity	Specificity
Functional MRI	59.2%	72.2%	45.4%
Structural MRI	60.1%	70.4%	49.1%
Personal Characteristic	59.6%	60.9%	58.3%
Structural MRI + Personal Characteristic	64.1%	75.7%	51.9%
Functional MRI + Personal Characteristic	65.0%	71.3%	58.3%

### Brain regions selected by machine learning

**Fig 9:**
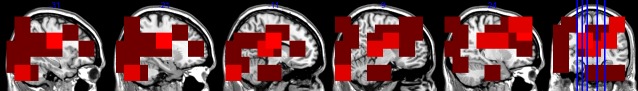
Extracted block regions from ADHD-200 structural MRI dataset. See text for details.

**Fig 10:**
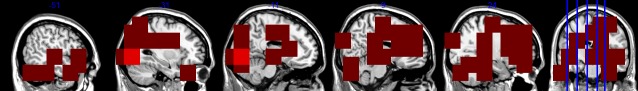
Extracted block regions from ABIDE fMRI dataset. See text for details.

We mapped the features found by MRMR to the brain space to examine which areas of the brain our trained classifiers used to diagnose ADHD and Autism. As mentioned before, each feature is a bin of a histogram, where each histogram belongs to a block of 16 × 16 × 16 voxels. MHPC identifies an effective combination of features, selected by MRMR, for extracting the blocks of brain that are important for diagnosis. In the ADHD-200 dataset, we mapped back the features selected from the analysis on structural images and personal characteristic data. In the ABIDE dataset, we mapped back the features selected from the analysis on the combination of functional images and personal characteristic data. These analyses were chosen as they yielded the highest accuracy values on the hold-out set. Figs 9 and 10 show the results. Dark red blocks are the regions that included only one selected feature, and the light red blocks are the blocks with two or more selected features. (Also see tables S7 to S10 Tables in the Supporting Information for more details of the selected regions.) Both analyses selected block regions that encompassed cortical, subcortical, and cerebellar brain regions throughout the brain. The other machine learning analyses we ran similarly selected block regions throughout the brain (results not shown).

## Discussion

The best two-class (ADHD versus healthy) imaging-based accuracy in the ADHD-200 dataset we could achieve was 69.6% using structural images and personal characteristic data, which was 7.1% better than the best imaging-based (functional scans, structural scans and personal characteristic data) diagnostic performance, 62.5%, achieved in the ADHD-200 global competition [6, 8]. (Note that our accuracy scores for the ADHD-200 test set did not include the 26 subjects from the Brown site, as their diagnostic labels have not been released). Sidhu *et al.* [9] achieved two-class accuracy on the ADHD-200 hold-out set of 66.7% using imaging and personal characteristic data. In a recent article, Dey *et al.* [36] achieved an accuracy of 73.6% on the test data, but they only used four of the imaging sites (including only 487 out of the 973 participants in the dataset) for their analysis. Chang *et al.* [24] achieved an accuracy of 70.0% using only male subjects from the ADHD-200 dataset (436 participants in total). They did not report results for female participants. The results of Dey *et al.* [36] and Chang *et al.* [24] are not comparable with our results since we used essentially all of the available subjects in the dataset for our analysis (excluding only a small number of participants for quality assurance reasons, see section Datasets).

For the ABIDE dataset we achieved an accuracy of 65.0% on a hold-out set, using functional images with personal characteristic data (note on this hold-out set, the baseline was 51.6%). This is better than the result of Nielsen *et al.* [12], who achieved 60.0% accuracy against their baseline of 53.6%. (The difference in baseline accuracies was because they omitted 148 of the individuals due to preprocessing problems.) Note that our results are not directly comparable to the results of Nielsen *et al.* [12] because their feature selection method (called “binning”) was run on the dataset a few times (with a leave-one-out scheme each time) using different numbers of “bins” (brain connections). Their reported best accuracy was actually based on examining the test set scores of all of the bins. That is, it was based indirectly on all of the data (not just the training set). This means that their true generalization accuracy may be under the reported 60.0% if run on a *hold-out* set. Katuwal et al. [15] achieved an accuracy of 67% classifying patients vs. controls using a combination of structural MRI data and age and IQ data from the ABIDE dataset. They included a subset of ABIDE participants in their analysis: 373 male controls and 361 male patients from the ABIDE dataset, out of a total of 1112 participants in the ABIDE dataset.

The ADHD-200 and ABIDE datasets are imbalanced for certain personal characteristics features. For example, ADHD-200 has a ratio of 4:1 males:females for patients but a ratio of 1:1 for healthy controls. We tested classification performance after balancing for sex, age, and Full IQ score. Performance was above chance (60.9 to 63.3%, compared to 50.0% chance) using fMRI or structural MRI data in this balanced context. (See S1 Appendix in the Supporting Information for details.)

To our knowledge, our results are the best published to date using essentially the whole ADHD-200 dataset or whole ABIDE dataset. This is in contrast to studies that excluded large proportions of the participants based on scanning site or sex.

We also found that, when using personal characteristic data along with structural or functional images, MHPC chose models that required fewer features than ones based only on structural or functional images. As an example, when using only functional images, we achieved an accuracy of 59.7% with 469 features, but when using personal characteristic features as well as functional image features, the accuracy increased to 64.3% while using only 137 features in the ADHD-200 dataset; see Tables 5 and 11. We also observe in Figs 7 and 8 that MHPC chose models with fewer features when using personal characteristic data.

This report also shows that it is possible to diagnose either ADHD or autism with accuracy levels 14.5% and 12.5% above chance using structural MR brain images.

### Biological interpretation

There are multiple ways to analyze a labeled dataset. One approach—standard in biostatistics—is an “association study” analysis, which looks for group-level effects or differences, for example differences in mean activation in various brain regions between patients with ADHD and controls. Our analysis, however, is based on an alternative “machine learning classification” approach, which looks for patterns in the data that differentiate individuals of different classes, for example patients vs. controls. There are important differences in what one can conclude based on these two approaches. (Also see Leek and Peng [37].) Association studies often discover group-level differences even in the presence of substantial overlap among individuals from the groups. In this case, such group-level differences do not translate into features that can differentiate individuals with high or even moderate accuracy. There are limitations on what one can conclude from group-level differences in the presence of such overlap, in so far as mental illnesses are properties of individuals, not groups. (Also see discussion in Brown *et al.* [8].)

By contrast, a machine learning classification analysis attempts to learn patterns that can accurately differentiate individuals. In the current study, the goal is to determine a participant’s class (patients vs. healthy control). Along with the other machine learning analyses of ADHD and autism discussed above, the machine learning classifier analysis presented here demonstrates that there are patterns in structural MRI and resting state functional MRI data that can distinguish individual patients and healthy controls with a reasonable degree of accuracy.

Group-level comparisons have a different goal: to identify biological differences between patients and controls that might be related to the presence or absence of a disease [38–41]. Those studies almost universally ignore the possibility of overlap among patients and controls. Brown *et al.* [8] replicated previous demonstrations of group-level differences between patients with ADHD and controls in terms of the resting state default mode functional connectivity in posterior cingulate cortex. However, that study also showed that patients and controls exhibited very large overlap in their functional connectivity values for posterior cingulate cortex, as shown in Fig 5 of [8]. (Here, the default mode functional connectivity value for a region was defined as the mean weighting value, over voxels in the region, for the default mode network component identified by independent components analysis (ICA)). The value of this feature is, therefore, not sufficient to determine whether the associated subject is likely to be a patient or a control.

If two features both contain the same information regarding class membership, the learned classifier can omit one of them. For example, suppose a psychiatric disease is associated with identical changes in fMRI activation in two brain regions, A1 and A2. A learned classifier for that disease can include only one of the regions, say A1, and ignore the other region, A2, because A2 does not provide any *additional* information for performing the classification task. Importantly, this omission does not imply that A2 is not involved in the disease.

Extracting domain knowledge from a learned classifier is not straight-forward. There is not usually a simple relationship between the learned feature weights in a classifier model and potential underlying domain knowledge. (See [42] and Section 2.6 of [43] for discussion.) In particular, different feature weights usually cannot be considered independently of each other, and the magnitude of a feature’s weight does not map straight-forwardly to that feature’s role or lack thereof in class membership (see Fig 1 of [43]). Due to these difficulties, we report simply those regions of the MR image volume used by our classifiers for two-class classification of patients vs. controls. The image features used by our classifiers include large regions throughout the cortex, subcortical white matter and nuclei, and cerebellum. These regions include a large portion of the total brain volume. (See Figs 9 and 10 and tables S7 to S10 Tables in the Supporting Information.)

Changes in gray matter volume are associated with ADHD, particularly in frontal brain regions (see [38]). Gray matter volume changes are also associated with autism spectrum disorder, particularly in frontal and temporal regions, the amygdala, hippocampus, caudate and other basal ganglia nuclei, and the cerebellum (see [38, 39]). It has been suggested that patients with ADHD exhibit disrupted resting state fMRI activity patterns, particularly in the default mode network, sensorimotor network, attention network, striatum, and cerebellum (reviewed in [40]). Patients with autism are reported to exhibit reduced resting state functional connectivity in the default mode network (reviewed in [41]). Many of the regions used by our diagnostic classifiers overlap with brain regions previously associated with ADHD or autism. In addition, some regions are not typically associated with either disease. For example, changes in occipital cortex are not associated with ADHD. It may be possible that these regions play some hitherto unrecognized role in ADHD or autism, though replication of the findings reported here would be essential for further developing this line of reasoning.

### Large Datasets and Heterogeneity

The ADHD-200 and ABIDE datasets are large (973 and 1112 participants, respectively). As discussed in the Introduction, this offers important advantages. To assemble these large datasets, participants were combined from multiple sites (8 for ADHD-200, 17 for ABIDE). This approach introduces heterogeneity into the dataset in terms of differences among MRI scanners, data collection protocols and participant populations. In one sense, this heterogeneity is useful as we work toward a deployable clinical machine learned system because such a system must cope with heterogeneity in data across hospitals. However, the heterogeneity also brings up the fundamental issue of how we define ground truth. In this study, we use the diagnostic categories supplied by the creators of the ADHD-200 and ABIDE datasets as the ground truth labels that the classifiers must reproduce. The different institutions contributing to a given dataset (ADHD-200 or ABIDE) used somewhat different diagnostic criteria (see [5] and [10]). It is possible that the diagnostic labels are not entirely consistent due to these differences. This may be a contributing factor in the lower accuracy rates reported for the ADHD-200 and ABIDE datasets in comparison to smaller datasets (see Introduction for more details). One way to test the effects of heterogeneity in the diagnostic labels would be to collect a new, large neuroimaging dataset using identical clinical criteria at multiple institutions, which would require overcoming substantial logistical and political challenges. Until such a dataset is available, existing datasets like ADHD-200 and ABIDE provide the best means of testing automated classification of mental health disorders with input data from large neuroimaging datasets.

### Clinical Utility

Our long-term goal is to produce a clinically-useful classifier than can perform high-accuracy differential diagnosis using brain imaging data. The work reported here is a step toward that goal. The patient populations in most mental health clinics include very few healthy individuals because most healthy people do not seek help at those clinics. As such, the classifier reported here might not be directly useful for clinicians; however, our approach does provide important results in the basic science of inferring clinical information about individual patients from brain imaging data. We demonstrate that a single learner can produce significantly above-chance accuracy in the binary classification problem in two large brain imaging datasets with different diagnostic endpoint (ADHD and autism). Furthermore, we obtained the best accuracies reported to date, on both classification problems, using essentially all of the participants in the ADHD-200 and ABIDE datasets. While continued work will be necessary to achieve the goal of high-accuracy differential diagnosis with a classifier system using brain imaging data, the work reported here is a critical step, as it demonstrates the potential of this machine learning approach.

### Conclusions

To summarize, we have improved the results on classification of ADHD and autism on two large datasets, ADHD-200 and ABIDE, which show that there are important signals in the brain images that can distinguish ADHD (resp., autism) from controls, which can be extracted using appropriate preprocessing and learning algorithms. In particular, we define 3D analogues to the standard 2D HOG features, which are well-known for object detection, and show they can be useful for classification of psychiatric illnesses using brain images. Since we successfully applied our method to learn classifiers from two large multi-site datasets, we expect that our approach will also be able to produce tools that can effectively classify other psychiatric disorders, from structural and functional MRI data, and hope that this will lead to extensions that are clinically relevant for various diseases. Further research is needed to address these questions.

## Supporting Information

S1 Appendix Supporting information for analysis with balanced datasets. (PDF)

S1 Fig Age distribution in balanced datasets. The figures represent the age distribution for each of the balanced datasets (bADHD-200 and bABIDE, see S1 Appendix). For both datasets, the distribution is identical for the healthy control and patient groups because we created the balanced datasets so as to satisfy this criterion.
(TIF)

S2 Fig Full IQ score distribution in balanced datasets. The figures represent the Full IQ score distribution for each of the balanced datasets (bADHD-200 and bABIDE, see S1 Appendix). For both datasets, the distribution is identical for the healthy control and patient groups because we created the balanced datasets so as to satisfy this criterion.
(TIF)

S1 Table Results for bADHD-200 using personal characteristic data. See S1 Appendix for details.
(PDF)

S2 Table Results for bABIDE using personal characteristic data. See S1 Appendix for details.
(PDF)

S3 Table Results for bADHD-200 using functional image data. See S1 Appendix for details.
(PDF)

S4 Table Results for bABIDE using functional image data. See S1 Appendix for details.
(PDF)

S5 Table Results for bADHD-200 using structural image data. See S1 Appendix for details.
(PDF)

S6 Table Results for bABIDE using structural image data. See S1 Appendix for details.
(PDF)

S7 Table Block regions selected by machine learning as diagnostic for ADHD from ADHD-200 structural MRI data. Region names denote the brain region in which each block centre was located. Blocks were large, and some blocks fell within multiple different brain regions. Blocks whose centre did not fall inside any atlas region were assigned to the region whose centroid was closest to the block centre. Region names and locations are from the Harvard-Oxford Atlas (all non-cerebellar regions) and the Bangor Cerebellar Atlas (all cerebellar regions). X, Y, and Z are coordinates in mm of each block region’s centre in MNI space.
(PDF)

S8 Table Block regions selected by machine learning as diagnostic for ADHD from ADHD-200 fMRI data. Conventions as in S7 Table.
(PDF)

S9 Table Block regions selected by machine learning as diagnostic for autism from ABIDE structural MRI data. Conventions as in S7 Table.
(PDF)

S10 Table Block regions selected by machine learning as diagnostic for autism from ABIDE fMRI data. Conventions as in S7 Table.
(PDF)
