# Supplementary material for: Using Functional or Structural Magnetic Resonance Images and Personal Characteristic Data to Identify ADHD and Autism
Source: PLoS One. 2016 Dec 28;11(12):e0166934. doi: 10.1371/journal.pone.0166934 (PMC5193362; doi:10.1371/journal.pone.0166934)
Supplement: S1 Appendix — (PDF) [file pone.0166934.s001.pdf]

S1 Appendix. Supporting information for analysis with balanced datasets. The ADHD-200 and ABIDE datasets have imbalances in terms of certain personal characteristics (PC) features. For example, in the ADHD-200 dataset, the ratio of males to females is approximately 1:1 for controls and 4:1 for patients (see Tables 1 and 2 in the main text). (This ratio difference reflects the previously-reported observation that ADHD is more common in males than females [1].)

To explore the effects of the imbalance of PC features (i.e. non-imaging covariates), we produced balanced datasets – balanced ADHD-200 (bADHD-200) and balanced ABIDE (bABIDE) – by subsampling participants from the ADHD-200 and ABIDE datasets, respectively. The bADHD-200 dataset includes 450 participants (225 healthy controls and 225 patients), and the bABIDE dataset includes 728 participants (364 healthy controls and 364 patients). bADHD-200 and bABIDE are balanced for sex, age, and Full IQ score. Specifically, we binned participants from the ADHD-200 or ABIDE datasets based on sex (2 bins), age (10 bins), and Full IQ score (10 bins). The bADHD-200 and bABIDE datasets were created so as to guarantee that each bin has identical numbers of patients and controls. For example, the bADHD-200 dataset contains equal numbers of controls and patients who were female, aged 10.0-11.5 years, and had Full IQ score in the range 105-113 at the time of data collection. Distributions of age and Full IQ score are shown in figures S1 Fig and S2 Fig.

For both the bADHD-200 and bABIDE datasets, we performed classifier analysis using the MHCP algorithm to generate binary classifiers (patient vs. control). One fifth of each dataset was randomly selected as the hold-out set, and the remaining 4/5 of the data were used as the training set. As before, we ran 5-fold cross validation on the training set to select the best learner and its parameters. We then ran this learner and parameters on the whole training dataset, and we tested the resulting learned classifier on the hold-out set. We considered three experiments: (1) using only the balanced PC features (sex, age, Full IQ score), (2) using only the fMRI data, and (3) using only the structural MRI data. Note that chance accuracy is 50.0% for both balanced datasets because they contain equal numbers of patients and controls.

Results are shown in tables S1 Table to S6 Table. As expected, classifier analysis using only balanced PC features produced accuracies no better than chance (bADHD-200 45.6%, bABIDE 46.9% hold out accuracy, compared to 50.0% chance). Analyses using functional MRI or structural MRI data yielded hold-out accuracies above chance, in the range of 60.9% to 63.3% (see tables S1 Table to S6 Table). These results confirm that the classifier results reported in the main text are not based simply on imbalances in sex, age, or Full IQ score between the healthy control and patient groups.

References

1. Bauermeister JJ, Shrout PE, Chávez L, Rubio-Stipec M, Ramírez R, Padilla L, et al. ADHD and gender: are risks and sequela of ADHD the same for boys and girls? Journal of child psychology and psychiatry, and allied disciplines. 2007;48:831–839.
