# Supplementary figures and images for: Using Functional or Structural Magnetic Resonance Images and Personal Characteristic Data to Identify ADHD and Autism
Source: PLoS One. 2016 Dec 28;11(12):e0166934. doi: 10.1371/journal.pone.0166934 (PMC5193362; doi:10.1371/journal.pone.0166934)

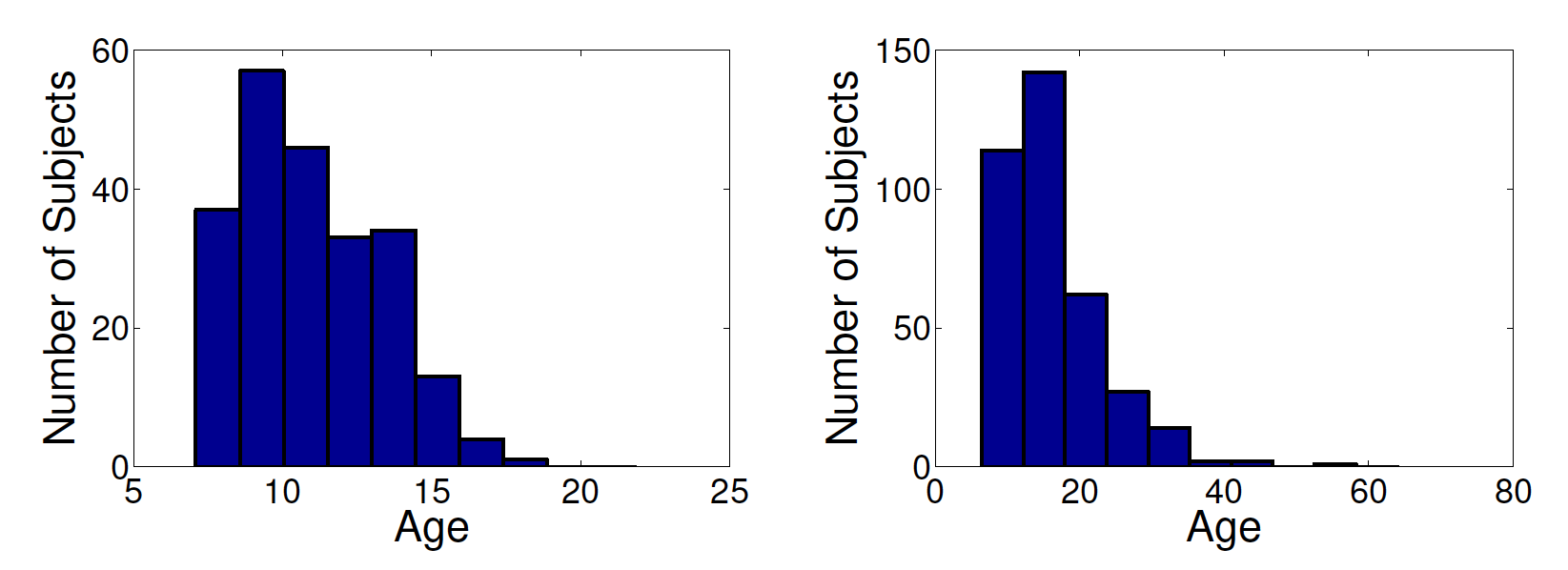

Supplement: S1 Fig — The figures represent the age distribution for each of the balanced datasets (bADHD-200 and bABIDE, see S1 Appendix). For both datasets, the distribution is identical for the healthy control and patient groups because we created the balanced datasets so as to satisfy this criterion. (TIF) [file pone.0166934.s002.tif]

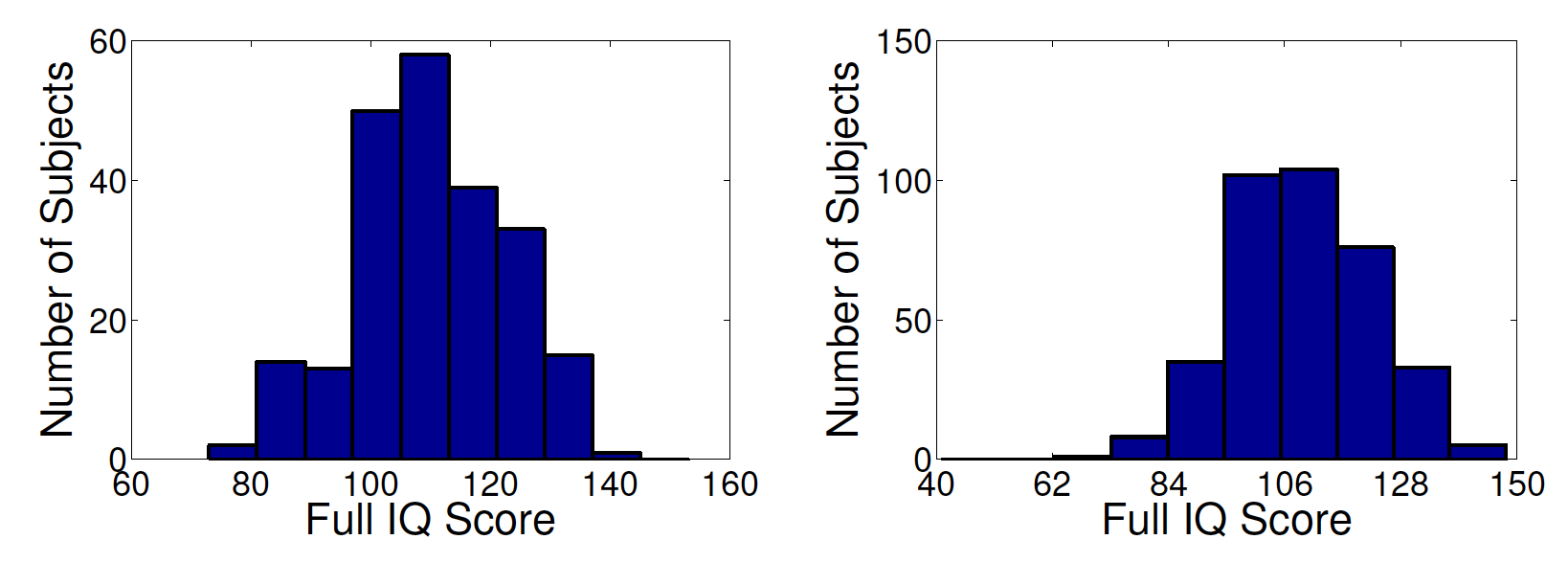

Supplement: S2 Fig — The figures represent the Full IQ score distribution for each of the balanced datasets (bADHD-200 and bABIDE, see S1 Appendix). For both datasets, the distribution is identical for the healthy control and patient groups because we created the balanced datasets so as to satisfy this criterion. (TIF) [file pone.0166934.s003.tif]
