# Supplementary material for: Using Functional or Structural Magnetic Resonance Images and Personal Characteristic Data to Identify ADHD and Autism
Source: PLoS One. 2016 Dec 28;11(12):e0166934. doi: 10.1371/journal.pone.0166934 (PMC5193362; doi:10.1371/journal.pone.0166934)
Supplement: S1 Table — See S1 Appendix for details. (PDF) [file pone.0166934.s004.pdf]

S1 Table. Results for bADHD-200, personal characteristic data. See S1 Appendix for details.

Learner, L	Number of features, $\lvert FS^*(L) \rvert$	Training Accuracy, $Eacc(L, D_{train}, FS^*(L))$	Range	Test Accuracy, $acc(L^*, D_{test}, FS^*(L^*))$
RBF-2	1	48.9%	2.5%	45.6%
RBF-3	1	48.9%	2.5%	
RBF-6	1	48.1%	2.5%	
RBF-8	2	48.1%	1.1%	
RBF-9	2	48.1%	5.2%	
