# Supplementary material for: Using Functional or Structural Magnetic Resonance Images and Personal Characteristic Data to Identify ADHD and Autism
Source: PLoS One. 2016 Dec 28;11(12):e0166934. doi: 10.1371/journal.pone.0166934 (PMC5193362; doi:10.1371/journal.pone.0166934)
Supplement: S2 Table — See S1 Appendix for details. (PDF) [file pone.0166934.s005.pdf]

S2 Table. Results for bABIDE, personal characteristic data. See S1 Appendix for details.

Learner, L	Number of features, $\|FS^*(L)\|$	Training Accuracy, $Eacc(L, D_{train}, FS^*(L))$	Range	Test Accuracy, $acc(L^*, D_{test}, FS^*(L^*))$
RBF-4	2	48.5%	11.6%	
RBF-3	2	48.4%	12.5%	
RBF-5	2	48.2%	12.5%	
RBF-7	3	47.7%	10.7%	46.9%
RBF-8	3	47.7%	12.5%	
