# Supplementary material for: Using Functional or Structural Magnetic Resonance Images and Personal Characteristic Data to Identify ADHD and Autism
Source: PLoS One. 2016 Dec 28;11(12):e0166934. doi: 10.1371/journal.pone.0166934 (PMC5193362; doi:10.1371/journal.pone.0166934)
Supplement: S3 Table — See S1 Appendix for details. (PDF) [file pone.0166934.s006.pdf]

S3 Table. Results for bADHD-200, functional image data. See S1 Appendix for details.

Learner, L	Number of features, $\lvert FS^*(L)\rvert$	Training Accuracy, $Eacc(L, D_{train}, FS^*(L))$	Range	Test Accuracy, $acc(L^*, D_{test}, FS^*(L^*))$
RBF-9	260	70.0%	11.1%	
RBF-8	210	70.0%	13.8%	
RBF-4	53	69.7%	13.8%	
RBF-6	252	69.7%	8.3%	
RBF-7	265	69.7%	6.9%	63.3%
