# Supplementary material for: Using Functional or Structural Magnetic Resonance Images and Personal Characteristic Data to Identify ADHD and Autism
Source: PLoS One. 2016 Dec 28;11(12):e0166934. doi: 10.1371/journal.pone.0166934 (PMC5193362; doi:10.1371/journal.pone.0166934)
Supplement: S4 Table — See S1 Appendix for details. (PDF) [file pone.0166934.s007.pdf]

S4 Table. Results for bABIDE, functional image data. See S1 Appendix for details.

Learner, L	Number of features, $\lvert FS^*(L)\rvert$	Training Accuracy, $Eacc(L, D_{train}, FS^*(L))$	Range	Test Accuracy, $acc(L^*, D_{test}, FS^*(L^*))$
RBF-4	65	60.4%	13.3%	
RBF-6	288	60.1%	17.6%	
RBF-3	18	59.6%	12.5%	
RBF-5	51	59.4%	22.2%	
RBF-9	360	59.4%	9.9%	63.0%
