# Supplementary material for: Using Functional or Structural Magnetic Resonance Images and Personal Characteristic Data to Identify ADHD and Autism
Source: PLoS One. 2016 Dec 28;11(12):e0166934. doi: 10.1371/journal.pone.0166934 (PMC5193362; doi:10.1371/journal.pone.0166934)
Supplement: S5 Table — See S1 Appendix for details. (PDF) [file pone.0166934.s008.pdf]

S5 Table. Results for bADHD-200, structural image data. See S1 Appendix for details.

Learner, L	Number of features, $\|FS^*(L)\|$	Training Accuracy, $Eacc(L, D_{train}, FS^*(L))$	Range	Test Accuracy, $acc(L^*, D_{test}, FS^*(L^*))$
RBF-4	97	66.3%	15.8%	
RBF-9	188	66.3%	13.8%	
RBF-7	158	66.1%	11.1%	
RBF-5	217	65.8%	11.1%	63.3%
RBF-8	170	65.8%	15.2%	
