# Supplementary material for: Using Functional or Structural Magnetic Resonance Images and Personal Characteristic Data to Identify ADHD and Autism
Source: PLoS One. 2016 Dec 28;11(12):e0166934. doi: 10.1371/journal.pone.0166934 (PMC5193362; doi:10.1371/journal.pone.0166934)
Supplement: S6 Table — See S1 Appendix for details. (PDF) [file pone.0166934.s009.pdf]

**S6 Table. Results for bABIDE, structural image data.** See S1 Appendix for details.

| Learner, L | Number of features, $\|FS^*(L)\|$ | Training Accuracy, $Eacc(L, D_{train}, FS^*(L))$ | Range | Test Accuracy, $acc(L^*, D_{test}, FS^*(L^*))$ |
|---|---|---|---|---|
| RBF-9 | 311 | 59.1% | 6.0% | |
| RBF-6 | 133 | 59.1% | 8.0% | |
| RBF-5 | 170 | 58.7% | 3.7% | 60.9% |
| RBF-3 | 61 | 58.7% | 12.3% | |
| RBF-7 | 166 | 58.5% | 9.4% | |
