# Supplementary material for: Using Functional or Structural Magnetic Resonance Images and Personal Characteristic Data to Identify ADHD and Autism
Source: PLoS One. 2016 Dec 28;11(12):e0166934. doi: 10.1371/journal.pone.0166934 (PMC5193362; doi:10.1371/journal.pone.0166934)
Supplement: S7 Table — Region names denote the brain region in which each block centre was located. Blocks were large, and some blocks fell within multiple different brain regions. Blocks whose centre did not fall inside any atlas region were assigned to the region whose centroid was closest to the block centre. Region names and locations are from the Harvard-Oxford Atlas (all non-cerebellar regions) and the Bangor Cerebellar Atlas (all cerebellar regions). X, Y, and Z are coordinates in mm of each block region’s centre in MNI space. (PDF) [file pone.0166934.s010.pdf]

S7 Table. Block regions for ADHD-200 structural image data. Part 1

Region	X	Y	Z
Left Cerebellum Crus II	3	-95	-33
Vermis Cerebellum Crus I	51	-95	-33
Left Cerebellum Crus I	-29	-79	-33
Left Inferior Temporal Gyrus temporooccipital part	-61	-63	-33
Left Cerebellum Crus I	-29	-63	-33
Right Cerebellum Crus I	51	-63	-33
Brain-Stem	3	-31	-33
Brain-Stem	3	-15	-33
Right Parahippocampal Gyrus anterior division	19	-15	-33
Right Inferior Temporal Gyrus posterior division	51	-15	-33
Left Temporal Pole	-61	17	-33
Left Temporal Pole	-45	33	-33
Left Temporal Pole	-61	49	-33
Right Temporal Pole	51	49	-33
Left Lateral Occipital Cortex inferior division	-61	-95	-17
Left Lateral Occipital Cortex inferior division	-45	-95	-17
Left Occipital Pole	-13	-95	-17
Right Occipital Pole	3	-95	-17
Left Cerebellum Crus I	-29	-79	-17
Left Cerebellum Crus I	-13	-79	-17
Right Cerebellum Crus I	35	-79	-17
Right Lateral Occipital Cortex inferior division	51	-79	-17
Left Temporal Occipital Fusiform Cortex	-45	-63	-17
Right Lateral Occipital Cortex inferior division	51	-63	-17
Left Inferior Temporal Gyrus temporooccipital part	-61	-47	-17
Left Middle Temporal Gyrus posterior division	-61	-15	-17
Left Inferior Frontal Gyrus pars triangularis	-61	49	-17
Left Frontal Pole	-45	49	-17
Left Occipital Pole	-29	-95	-1
Right Lateral Occipital Cortex inferior division	51	-95	-1
Left Occipital Fusiform Gyrus	-29	-79	-1
Left Lateral Occipital Cortex inferior division	-61	-63	-1
Right Lateral Occipital Cortex inferior division	51	-63	-1
Left Inferior Frontal Gyrus pars opercularis	-61	17	-1
Left Inferior Frontal Gyrus pars triangularis	-61	49	-1

Part 2

Region	X	Y	Z
Left Lateral Occipital Cortex inferior division	-61	-95	15
Left Lateral Occipital Cortex inferior division	-45	-95	15
Left Occipital Pole	-29	-95	15
Right Occipital Pole	3	-95	15
Right Occipital Pole	35	-95	15
Right Lateral Occipital Cortex inferior division	51	-95	15
Right Lateral Occipital Cortex superior division	51	-79	15
Right Lateral Occipital Cortex inferior division	51	-63	15
Left Cerebral White Matter	-29	-47	15
Left Planum Temporale	-61	-31	15
Right Cerebral White Matter	3	-31	15
Left Thalamus	-13	-15	15
Right Cingulate Gyrus anterior division	3	33	15
Left Frontal Pole	-29	49	15
Right Frontal Pole	35	49	15
Right Lateral Occipital Cortex superior division	51	-95	31
Left Lateral Occipital Cortex superior division	-45	-79	31
Left Cuneal Cortex	-13	-79	31
Right Cuneal Cortex	3	-79	31
Right Lateral Occipital Cortex superior division	35	-79	31
Right Lateral Occipital Cortex superior division	51	-79	31
Left Lateral Occipital Cortex superior division	-45	-63	31
Left Superior Parietal Lobule	-29	-47	31
Left Cingulate Gyrus posterior division	-13	-47	31
Left Cerebral White Matter	-13	-31	31
Right Cingulate Gyrus posterior division	3	-31	31
Right Cerebral White Matter	35	-31	31
Left Cerebral White Matter	-13	-15	31
Right Cerebral White Matter	19	1	31
Right Cerebral White Matter	19	17	31
Left Inferior Frontal Gyrus pars triangularis	-61	33	31
Left Middle Frontal Gyrus	-45	33	31
Left Inferior Frontal Gyrus pars triangularis	-61	49	31
Left Inferior Frontal Gyrus pars triangularis	-45	49	31

Part 3

Region	X	Y	Z
Left Lateral Occipital Cortex superior division	-61	-95	47
Left Lateral Occipital Cortex superior division	-29	-79	47
Right Lateral Occipital Cortex superior division	19	-79	47
Left Lateral Occipital Cortex superior division	-45	-63	47
Left Postcentral Gyrus	-61	-15	47
Right Cingulate Gyrus posterior division	3	-15	47
Right Cerebral White Matter	19	-15	47
Right Juxtapositional Lobule Cortex	3	1	47
Right Superior Frontal Gyrus	19	1	47
Left Middle Frontal Gyrus	-29	17	47
Right Middle Frontal Gyrus	35	17	47
Left Middle Frontal Gyrus	-45	33	47
Right Middle Frontal Gyrus	35	33	47
Right Middle Frontal Gyrus	51	33	47
Left Middle Frontal Gyrus	-61	49	47
Right Frontal Pole	19	49	47
Right Middle Frontal Gyrus	35	49	47
Left Lateral Occipital Cortex superior division	-61	-95	63
Right Lateral Occipital Cortex superior division	19	-95	63
Left Lateral Occipital Cortex superior division	-61	-79	63
Left Lateral Occipital Cortex superior division	-29	-79	63
Right Lateral Occipital Cortex superior division	51	-79	63
Left Supramarginal Gyrus posterior division	-61	-63	63
Right Supramarginal Gyrus posterior division	51	-47	63
Left Postcentral Gyrus	-45	-31	63
Right Postcentral Gyrus	51	-31	63
Right Middle Frontal Gyrus	35	17	63
Right Middle Frontal Gyrus	51	17	63
Left Middle Frontal Gyrus	-45	33	63
Right Superior Frontal Gyrus	19	33	63
Right Superior Frontal Gyrus	35	33	63
Right Superior Frontal Gyrus	19	49	63
Right Middle Frontal Gyrus	35	49	63
Right Middle Frontal Gyrus	51	49	63
