# Supplementary material for: Using Functional or Structural Magnetic Resonance Images and Personal Characteristic Data to Identify ADHD and Autism
Source: PLoS One. 2016 Dec 28;11(12):e0166934. doi: 10.1371/journal.pone.0166934 (PMC5193362; doi:10.1371/journal.pone.0166934)
Supplement: S8 Table — Conventions as in S7 Table. (PDF) [file pone.0166934.s011.pdf]

S8 Table. Block regions for ADHD-200 functional image data. Part 1

Region	X	Y	Z
Right Cerebellum Crus I	-29	-95	-33
Left Cerebellum Crus I	-61	-79	-33
Left Cerebellum VI	-13	-63	-33
Vermis Cerebellum VIIIa	3	-63	-33
Left Cerebellum VI	-29	-47	-33
Right Cerebellum VIIIb	-13	-47	-33
Vermis Cerebellum IX	19	-47	-33
Right Cerebellum VI	35	-47	-33
Left Inferior Temporal Gyrus posterior division	-61	-31	-33
Left Cerebellum V	-29	-31	-33
Brain-Stem	-13	-31	-33
Right Cerebellum I-IV	19	-31	-33
Left Parahippocampal Gyrus anterior division	-29	-15	-33
Brain-Stem	-13	-15	-33
Left Parahippocampal Gyrus anterior division	-29	1	-33
Left Subcallosal Cortex	-13	17	-33
Left Frontal Medial Cortex	-29	49	-33
Left Lateral Occipital Cortex inferior division	-45	-79	-17
Vermis Cerebellum VI	3	-79	-17
Left Cerebellum VI	-29	-63	-17
Right Cerebellum V	3	-63	-17
Left Inferior Temporal Gyrus temporooccipital part	-45	-47	-17
Left Parahippocampal Gyrus posterior division	-29	-31	-17
Right Temporal Fusiform Cortex posterior division	35	-31	-17
Left Superior Temporal Gyrus anterior division	-45	-15	-17
Right Middle Temporal Gyrus posterior division	51	-15	-17
Left Planum Polare	-45	1	-17
Left Parahippocampal Gyrus anterior division	-29	1	-17
Left Cerebral Cortex	-13	1	-17
Right Parahippocampal Gyrus anterior division	19	1	-17
Right Temporal Pole	51	17	-17
Right Lingual Gyrus	3	-79	-1
Right Lateral Occipital Cortex inferior division	51	-79	-1
Left Lingual Gyrus	-29	-63	-1
Right Lingual Gyrus	19	-63	-1
Left Superior Temporal Gyrus posterior division	-45	-31	-1
Left Cerebral White Matter	-29	-31	-1
Right Insular Cortex	35	-15	-1
Right Superior Temporal Gyrus posterior division	51	-15	-1
Left Pallidum	-13	1	-1
Right Cerebral White Matter	3	1	-1
Right Subcallosal Cortex	3	17	-1
Right Cerebral White Matter	19	17	-1
Left Cerebral White Matter	-13	33	-1

Part 2

Region	X	Y	Z
Right Cingulate Gyrus anterior division	3	33	-1
Left Frontal Pole	-29	49	-1
Left Paracingulate Gyrus	-13	49	-1
Right Intracalcarine Cortex	19	-79	15
Right Lateral Occipital Cortex superior division	51	-79	15
Left Cerebral White Matter	-29	-63	15
Right Supracalcarine Cortex	19	-63	15
Right Lateral Occipital Cortex inferior division	51	-63	15
Left Supramarginal Gyrus posterior division	-45	-47	15
Left Cerebral White Matter	-29	-47	15
Left Parietal Operculum Cortex	-45	-31	15
Right Planum Temporale	35	-31	15
Right Planum Temporale	51	-31	15
Right Insular Cortex	35	-15	15
Right Central Opercular Cortex	51	-15	15
Left Central Opercular Cortex	-45	1	15
Left Insular Cortex	-29	1	15
Left Lateral Ventrical	-13	17	15
Left Cingulate Gyrus anterior division	-13	33	15
Right Cingulate Gyrus anterior division	3	33	15
Right Frontal Pole	35	33	15
Left Frontal Pole	-45	49	15
Right Frontal Pole	35	49	15
Right Lateral Occipital Cortex superior division	35	-63	31
Left Supramarginal Gyrus anterior division	-45	-31	31
Right Parietal Operculum Cortex	51	-31	31
Left Postcentral Gyrus	-45	-15	31
Left Cerebral White Matter	-29	-15	31
Right Cerebral White Matter	35	-15	31
Left Middle Frontal Gyrus	-45	17	31
Left Middle Frontal Gyrus	-29	17	31
Left Middle Frontal Gyrus	-29	33	31
Left Inferior Frontal Gyrus pars triangularis	-45	49	31
Left Lateral Occipital Cortex superior division	-29	-79	47
Right Precuneous Cortex	3	-63	47
Left Supramarginal Gyrus posterior division	-45	-47	47
Left Precuneous Cortex	-13	-47	47
Right Angular Gyrus	51	-47	47

Part 3

Region	X	Y	Z
Left Postcentral Gyrus	-29	-31	47
Right Postcentral Gyrus	35	-31	47
Left Postcentral Gyrus	-61	-15	47
Right Precentral Gyrus	35	-15	47
Right Postcentral Gyrus	51	-15	47
Left Middle Frontal Gyrus	-61	1	47
Left Middle Frontal Gyrus	-29	1	47
Right Superior Frontal Gyrus	19	17	47
Right Middle Frontal Gyrus	51	17	47
Left Middle Frontal Gyrus	-61	33	47
Right Middle Frontal Gyrus	35	33	47
Right Middle Frontal Gyrus	51	33	47
Right Middle Frontal Gyrus	35	49	47
Right Lateral Occipital Cortex superior division	19	-79	63
Left Superior Parietal Lobule	-45	-47	63
Left Superior Parietal Lobule	-29	-47	63
Right Precuneous Cortex	3	-47	63
Left Postcentral Gyrus	-45	-31	63
Left Postcentral Gyrus	-29	-31	63
Left Precentral Gyrus	-13	-31	63
Right Postcentral Gyrus	19	-31	63
Right Postcentral Gyrus	35	-31	63
Left Precentral Gyrus	-29	-15	63
Right Precentral Gyrus	35	-15	63
Left Cerebral Cortex	-45	1	63
Left Superior Frontal Gyrus	-13	1	63
Right Superior Frontal Gyrus	19	1	63
Right Middle Frontal Gyrus	35	1	63
Right Precentral Gyrus	51	1	63
Left Superior Frontal Gyrus	-13	17	63
Right Superior Frontal Gyrus	19	17	63
Right Middle Frontal Gyrus	35	17	63
Right Middle Frontal Gyrus	51	17	63
Right Superior Frontal Gyrus	3	33	63
