# Supplementary material for: Using Functional or Structural Magnetic Resonance Images and Personal Characteristic Data to Identify ADHD and Autism
Source: PLoS One. 2016 Dec 28;11(12):e0166934. doi: 10.1371/journal.pone.0166934 (PMC5193362; doi:10.1371/journal.pone.0166934)
Supplement: S9 Table — Conventions as in S7 Table. (PDF) [file pone.0166934.s012.pdf]

S9 Table. Block regions for ABIDE structural image data. Part 1

Region	X	Y	Z
Left Cerebellum Crus II	-13	-95	-33
Right Cerebellum Crus I	51	-47	-33
Brain-Stem	-13	-31	-33
Left Temporal Pole	-45	33	-33
Left Frontal Medial Cortex	-29	49	-33
Left Occipital Pole	-29	-95	-17
Left Occipital Pole	-13	-95	-17
Right Occipital Pole	3	-95	-17
Right Lateral Occipital Cortex inferior division	51	-95	-17
Right Cerebellum VI	35	-63	-17
Left Middle Temporal Gyrus anterior division	-61	1	-17
Left Frontal Medial Cortex	-13	33	-17
Left Occipital Fusiform Gyrus	-29	-79	-1
Right Lateral Occipital Cortex inferior division	51	-79	-1
Left Inferior Frontal Gyrus pars opercularis	-61	17	-1
Right Inferior Frontal Gyrus pars opercularis	51	17	-1
Left Occipital Pole	-13	-95	15
Right Occipital Pole	3	-95	15
Left Lateral Occipital Cortex superior division	-29	-79	15
Right Lateral Occipital Cortex superior division	35	-79	15
Left Thalamus	-13	-15	15
Right Cerebral White Matter	19	-15	15
Left Caudate	-13	1	15
Right Cingulate Gyrus anterior division	3	33	15
Right Frontal Pole	35	49	15
Left Lateral Occipital Cortex superior division	-61	-95	31
Right Occipital Pole	3	-95	31
Left Cuneal Cortex	-13	-79	31
Right Cuneal Cortex	19	-79	31
Left Lateral Occipital Cortex superior division	-45	-63	31
Right Lateral Occipital Cortex superior division	51	-63	31
Left Cerebral White Matter	-13	-31	31
Left Cerebral White Matter	-13	-15	31
Right Middle Frontal Gyrus	51	33	31
Right Frontal Pole	35	49	31
Right Inferior Frontal Gyrus pars triangularis	51	49	31
Left Lateral Occipital Cortex superior division	-45	-95	47
Right Lateral Occipital Cortex superior division	51	-95	47
Left Lateral Occipital Cortex superior division	-61	-79	47
Left Supramarginal Gyrus anterior division	-61	-31	47
Left Postcentral Gyrus	-61	-15	47
Right Postcentral Gyrus	51	-15	47
Right Middle Frontal Gyrus	35	1	47
Right Middle Frontal Gyrus	51	17	47

Part 2.

Region	X	Y	Z
Left Lateral Occipital Cortex superior division	-29	-95	63
Right Lateral Occipital Cortex superior division	51	-79	63
Left Supramarginal Gyrus posterior division	-61	-63	63
Left Postcentral Gyrus	-13	-47	63
Left Postcentral Gyrus	-61	-15	63
Right Precentral Gyrus	35	-15	63
Left Middle Frontal Gyrus	-61	17	63
Right Superior Frontal Gyrus	3	17	63
Left Middle Frontal Gyrus	-45	33	63
