# Supplementary material for: Using Functional or Structural Magnetic Resonance Images and Personal Characteristic Data to Identify ADHD and Autism
Source: PLoS One. 2016 Dec 28;11(12):e0166934. doi: 10.1371/journal.pone.0166934 (PMC5193362; doi:10.1371/journal.pone.0166934)
Supplement: S10 Table — Conventions as in S7 Table. (PDF) [file pone.0166934.s013.pdf]

S10 Table. Block regions for ABIDE functional image data. Part 1

Region	X	Y	Z
Right Cerebellum Crus II	19	-79	-33
Left Cerebellum Crus I	-45	-63	-33
Left Inferior Temporal Gyrus posterior division	-61	-31	-33
Right Cerebellum VI	35	-31	-33
Left Temporal Pole	-61	17	-33
Left Frontal Orbital Cortex	-29	33	-33
Vermis Cerebellum VI	3	-79	-17
Right Cerebellum VI	35	-63	-17
Right Lateral Occipital Cortex inferior division	51	-63	-17
Right Superior Temporal Gyrus anterior division	51	1	-17
Left Frontal Orbital Cortex	-45	33	-17
Right Frontal Pole	35	49	-17
Left Occipital Pole	-13	-95	-1
Left Lingual Gyrus	-13	-79	-1
Right Thalamus	19	-31	-1
Right Superior Temporal Gyrus posterior division	51	-31	-1
Left Heschl's Gyrus (includes H1 and H2)	-45	-15	-1
Right Thalamus	3	-15	-1
Right Planum Polare	51	1	-1
Left Inferior Frontal Gyrus pars triangularis	-61	33	-1
Left Inferior Frontal Gyrus pars triangularis	-45	33	-1
Right Inferior Frontal Gyrus pars triangularis	35	33	-1
Left Intracalcarine Cortex	-13	-79	15
Right Cerebral White Matter	3	17	15
Right Cerebral White Matter	19	17	15
Right Cerebral White Matter	19	33	15
Right Frontal Pole	19	49	15
Right Occipital Pole	19	-95	31
Left Lateral Occipital Cortex superior division	-29	-63	31
Left Cerebral White Matter	-29	-31	31
Right Cerebral White Matter	19	-31	31
Left Precentral Gyrus	-29	1	31
Right Precentral Gyrus	51	1	31
Right Cerebral White Matter	19	17	31
Left Lateral Occipital Cortex superior division	-13	-79	47
Right Cingulate Gyrus posterior division	3	-15	47
Right Juxtapositional Lobule Cortex	3	1	47
Right Middle Frontal Gyrus	35	17	47
Right Middle Frontal Gyrus	51	17	47
Right Superior Frontal Gyrus	19	33	47
Right Frontal Pole	19	49	47
Left Lateral Occipital Cortex superior division	-29	-63	63
Right Precentral Gyrus	19	-15	63
Right Precentral Gyrus	35	-15	63
